# Enhanced submucosal visualization using amber-red color imaging during gel-immersion colorectal endoscopic submucosal dissection

**DOI:** 10.1055/a-2749-3277

**Published:** 2025-12-17

**Authors:** Naohisa Yoshida, Ken Inoue, Osamu Dohi, Hardesh Dhillon, Reo Kobayashi, Yuri Tomita, Tomohisa Takagi

**Affiliations:** 1 Department of Molecular Gastroenterology and Hepatology, Graduate School of Medical Science, Kyoto Prefectural University of Medicine, Kyoto, Japan; 2 Department of Gastroenterology, Barwon Health, Victoria, Australia; 3 Department of Gastroenterology, Hakuaikai Hospital, Kyoto, Japan

Ensuring a safe and effective colorectal endoscopic submucosal dissection (ESD) requires prevention of perioperative bleeding, muscle injury and prompt hemostasis. Gel-immersion endoscopy has been reported to facilitate the identification of bleeding points by improving visualization 1 . In addition, we recently demonstrated that gel-immersion ESD (GI-ESD) can reduce muscle layer exposure and postoperative inflammatory response in duodenal ESD 2 .

A novel light-emitting diode (LED) endoscopic system (EP-8000; Fujifilm, Tokyo, Japan), released in 2024 and equipped with a new observation mode called amber-red color imaging (ACI), provides enhanced brightness and reduced halation 3 . ACI improves the visualization of vessels and the submucosal layer, thereby facilitating safer and more precise dissection with reduced perioperative bleeding 4 5 .

In the present case, an 81-year-old-man presented with a 30-mm Paris IIa laterally spreading lesion in the transverse colon ( Fig. 1 **a** , Video 1 ). Blue light imaging revealed an irregular surface and a vessel pattern consistent with high-grade dysplasia ( Fig. 1 **b** ). After completing marking and submucosal injection using 0.2% hyaluronic acid (Boston Scientific, MA, USA) with indigocarmine (final concentration: 0.003%), a full circumferential mucosal incision was performed using a Clutch Cutter (Fujifilm, Tokyo, Japan). A SureClip Traction Band (Micro-Tech, Nanjing, China) was then deployed to expose the submucosal layer. However, moderate fibrosis impeded clear visualization of the submucosal layer, underlying vessels, and muscle layer ( Fig. 2 **a** , Video 1 ). Using ACI under gel-immersion (Viscoclear, Otsuka Pharmaceuticals, Tokushima, Japan), visualization of them markedly improved compared with white-light imaging, providing enhanced magnification, effectively eliminating halation, and creating a natural submucosal buoyancy ( Fig. 2 **b, c** ). These advantages enabled precise and safe submucosal dissection. During episodes of minor bleeding, the bleeding point was easily identified owing to gel retention, allowing immediate hemostasis to be achieved ( Fig. 2 **d** ). The lesion was successfully resected en-bloc without significant bleeding or muscle injury (procedure time: 35 minutes). The histopathological diagnosis was high-grade adenoma with negative margins.

**Fig. 1:**
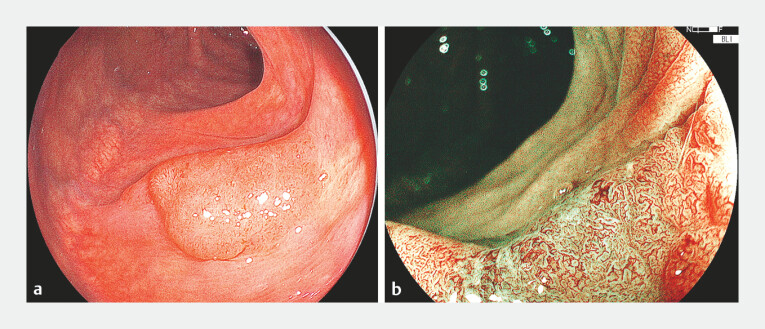
A case of colonic ESD. **a** An 81-year-old-man presented with a 30-mm Paris IIa laterally spreading lesion in the transverse colon. **b** Blue light imaging revealed an irregular surface and vessel pattern consistent with high-grade dysplasia. ESD, endoscopic submucosal dissection.

Enhanced submucosal visualization using ACI during gel-immersion colorectal ESD. During colonic ESD, ACI improved the visibility of vessels, submucosa, and muscle layer and ACI under gel provided a slightly magnified, halation-free view, enabling accurate submucosal dissection. ACI, amber-red color imaging; ESD, endoscopic submucosal dissection.Video 1

**Fig. 2:**
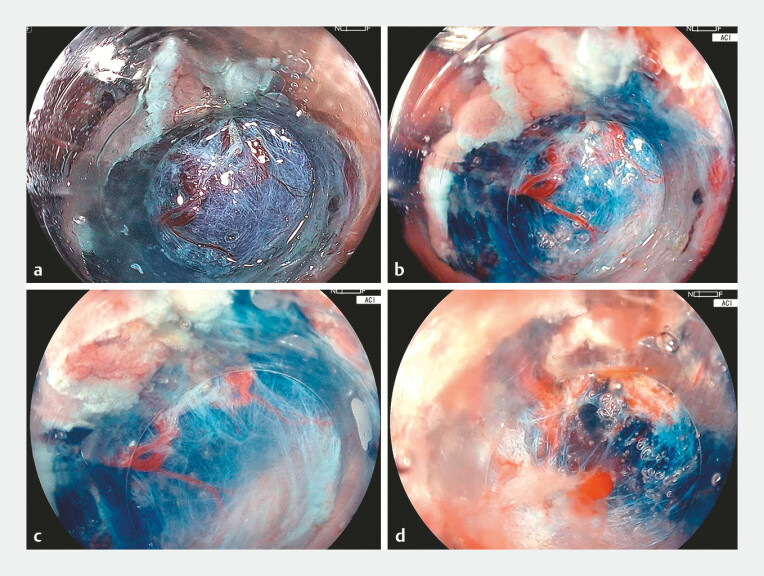
The efficacy of ACI in colonic ESD. **a** Moderate fibrosis obscured the visibility of vessels, submucosa and muscle layer. **b** ACI improved the visibility of vessels, submucosa, and muscle layer compared with white-light imaging. **c** ACI under gel provided a slightly magnified, halation-free view, enabling accurate and quick submucosal dissection. **d** In minor perioperative bleeding, the bleeding point was clearly detected owing to the gel’s retention effect, allowing immediate hemostasis. ACI, amber-red color imaging; ESD, endoscopic submucosal dissection.

In conclusion, the combination of ACI with gel-immersion ESD enhanced submucosal visualization, enabling safe and efficient dissection during colorectal ESD.

Endoscopy_UCTN_Code_TTT_1AQ_2AD_3AD

## Notes

Endoscopy E-Videos https://eref.thieme.de/e-videos
*E-Videos* is an open access online section of the journal *Endoscopy* , reporting on interesting cases and new techniques in gastroenterological endoscopy. All papers include a high-quality video and are published with a Creative Commons CC-BY license. Endoscopy E-Videos qualify for HINARI discounts and waivers and eligibility is automatically checked during the submission process. We grant 100% waivers to articles whose corresponding authors are based in Group A countries and 50% waivers to those who are based in Group B countries as classified by Research4Life (see: https://www.research4life.org/access/eligibility/ ). This section has its own submission website at https://mc.manuscriptcentral.com/e-videos .
